# Establishing a comprehensive list of mental health-related services and resource use items in Austria: A national-level, cross-sectoral country report for the PECUNIA project

**DOI:** 10.1371/journal.pone.0262091

**Published:** 2022-01-21

**Authors:** Claudia Fischer, Susanne Mayer, Nataša Perić, Judit Simon, on behalf of the PECUNIA Group

**Affiliations:** 1 Department of Health Economics, Center for Public Health, Medical University of Vienna, Vienna, Austria; 2 Department of Psychiatry, Warneford Hospital, University of Oxford, Oxford, United Kingdom; University of Thessaly, GREECE

## Abstract

**Background:**

A comprehensive, comparable assessment of the economic disease burden and the value of relevant care forms a major challenge in the case of mental diseases. This study aimed to inform the development of a resource use measurement (RUM) instrument and harmonized reference unit costs valid for multi-sectoral and multi-national cost assessments for mental health diseases as part of the European PECUNIA project.

**Methods:**

An iterative, multi-methods approach was applied. Systematic literature reviews appended with national grey literature searches in six European countries were conducted to generate preliminary, literature-based, international, mental health-related service and resource use lists for all investigated sectors in 2018. As part of a multi-national expert survey, these lists were reviewed by 18 Austrian sector-specific experts regarding the clarity, relevance, comprehensiveness and availability in the Austrian context.

**Results:**

Out of 295 items included in the preliminary, international, sector-specific lists (health and social care—201 items, criminal justice—35 items, education—39 items; patient, family and informal care—20 items), a total of 261 items and descriptions (88%) were considered clear by all experts. 42 items (14%) were considered not existing in Austria, and 111 items (38%) were prioritized regarding their relevance in the national context. Thirteen additional items (4%) were suggested to be added to accommodate for Austria-specific features of the individual sectors. Major typological difficulties based on item names were observed.

**Conclusions:**

The identified country-specific variations and general typological bias and their potential contributions to service and resource use cost variations across countries and sectors call for further systematic investigation. Next, PECUNIA will develop internationally harmonized and comparable definitions of the listed items and their units of analysis based on a new conceptual multi-sectoral costing framework. The developed lists will require consolidation and further prioritization for the development of a patient-reported RUM instrument and consequent reference unit cost valuation.

## Introduction

A comprehensive and comparable assessment of the economic burden of a disease and the value of relevant care in economic evaluations across sectors and countries are in general difficult, however, it is especially of a challenge in the case of mental diseases [1]. One factor is the reliability of the measurement and the different thresholds regarding the epidemiology of diseases [2,3]. Even minor changes in definitions may cause considerable variation in the estimation of disease prevalence and service utilization [4,5]. Another factor is that although the economic burden of mental diseases in regards to costs is thought to be massive [6], resulting in doubled total costs for persons with mental diseases compared to those without [7], calculating exact costs is complicated [8,9].

This is particularly the case if health care provision and funding are both fragmented, such as in Austria [10,11]. In the Austrian health care system, the principal responsibility for the public health system is shared by the central government, nine federal state governments and agents. There is no gate-keeping system in place, which means that medical services and treatments can be utilized to a great extent and with great variety across different regions [10,12]. Around 37% of Austrians have complementary private health insurance [13], resulting in a two-tier healthcare system.

For many ill-health conditions, such as mental diseases, the economic consequences are not limited to the healthcare sector, but also spread to other sectors [14]. These interventions within the healthcare sector, which have consequences spilling over to other sectors, are referred to as inter-sectoral costs and benefits (ICBs) [15]. Relevant sectors include the social sector (e.g. through social care), the criminal justice system (e.g. through police interventions), the education sector (e.g. through special education needs), and the patient and family domains (e.g. due to informal caregiving activities). Next to direct cost consequences (e.g. service costs, out-of-pocket costs) and tangible consequences that can be valued in monetary terms (e.g. changes in productivity, care giving or in the level of vandalism), also the consideration of non-monetary, intangible consequences (e.g. stigma) is crucial in the case of mental health. By considering all these types of ICBs, the perspective from which an economic evaluation is conducted will be comprehensive enough to form a valid decision base for decision makers and qualifies to be categorized as the perspective type that is broadest and most advocated, which is the societal perspective. [16]

Internationally, there have been different attempts to assess the costs of mental diseases in the healthcare sector. Nevertheless, health economic studies often adopt the healthcare sector perspective in their analysis and do not include costs affecting sectors beyond [e.g. [17,18]]. In a recent review of published health economic evaluations in Austria, only one study could be identified that included costs from the criminal justice sector [19]. This might be due to the lack of available data and valuation methods [20]. At the same time, these inter-sectoral costs were found to contribute a considerable proportion to the total costs of mental diseases [7,15,21,22]. With economic evaluations being increasingly used as a base for decision making in healthcare, a comprehensive reflection of the societal costs associated with a disease is also vital in this context and has been already recommended in national health economics guidelines in the Netherlands and Spain [23,24]. While intangible consequences so far lack monetary considerations in economic evaluations, differences in costing methodologies for direct costs and tangible consequences between studies and countries often result in incomparable (unit) cost estimates [25–28]. Developing methodology to tackle the latter problem in practice was the main focus of the EU project PECUNIA, i.e. the ProgrammE in Costing, resource-use measurement and outcome valuation for Use in multisectoral National and International health economic evaluAtions. PECUNIA aims to establish standardised costing and outcome assessment systems that directly enable comparability, applicability and transferability of cost-effectiveness evidence for health-related interventions across sectors and countries [29,30]. The streams of work focused on the assessment of costs across multiple sectors related to health and social care (HCSC), criminal justice (CJ), education (ED), employment and productivity (EP), and patient, family and informal care (PFI) alongside four horizontal methodological axes following the steps of identification, definition, measurement and valuation of resources in the relevant work streams. Further information on the PECUNIA project can be found elsewhere [29–31].

The aim of this study was to synthesize international and national information on relevant services related to mental diseases in general, and three specific disease areas (depression, schizophrenia and post-traumatic stress disorder (PTSD)) across multiple sectors, and subject it to expert assessment in Austria. This will inform the development of a patient-reported resource use measurement (RUM) instrument targeted at adults and adolescents and harmonized reference unit costs valid for multi-sectoral and multi-national cost assessments as part of the European PECUNIA project.

## Methods

This study was conducted as part of the first identification step in PECUNIA, which aimed at collating mental health-related services and resource use items within the relevant sectors for Europe using six selected countries (Austria, Germany, Hungary, the Netherlands, Spain, the United Kingdom). Methods followed a cross-sectoral and cross-country harmonized approach similar to earlier research conducted for Germany [8]. For the employment and productivity sector, a systematic literature review of measurement instruments of productivity loss of paid and unpaid work was conducted. In consequence, these instruments underwent a newly designed appraisal framework to assess their content validity and suitability in terms of availability, feasibility, and applicability for their use in economic evaluations from a societal perspective. The methodology of this adopted approach is covered in detail elsewhere [32].

**Fig 1:**
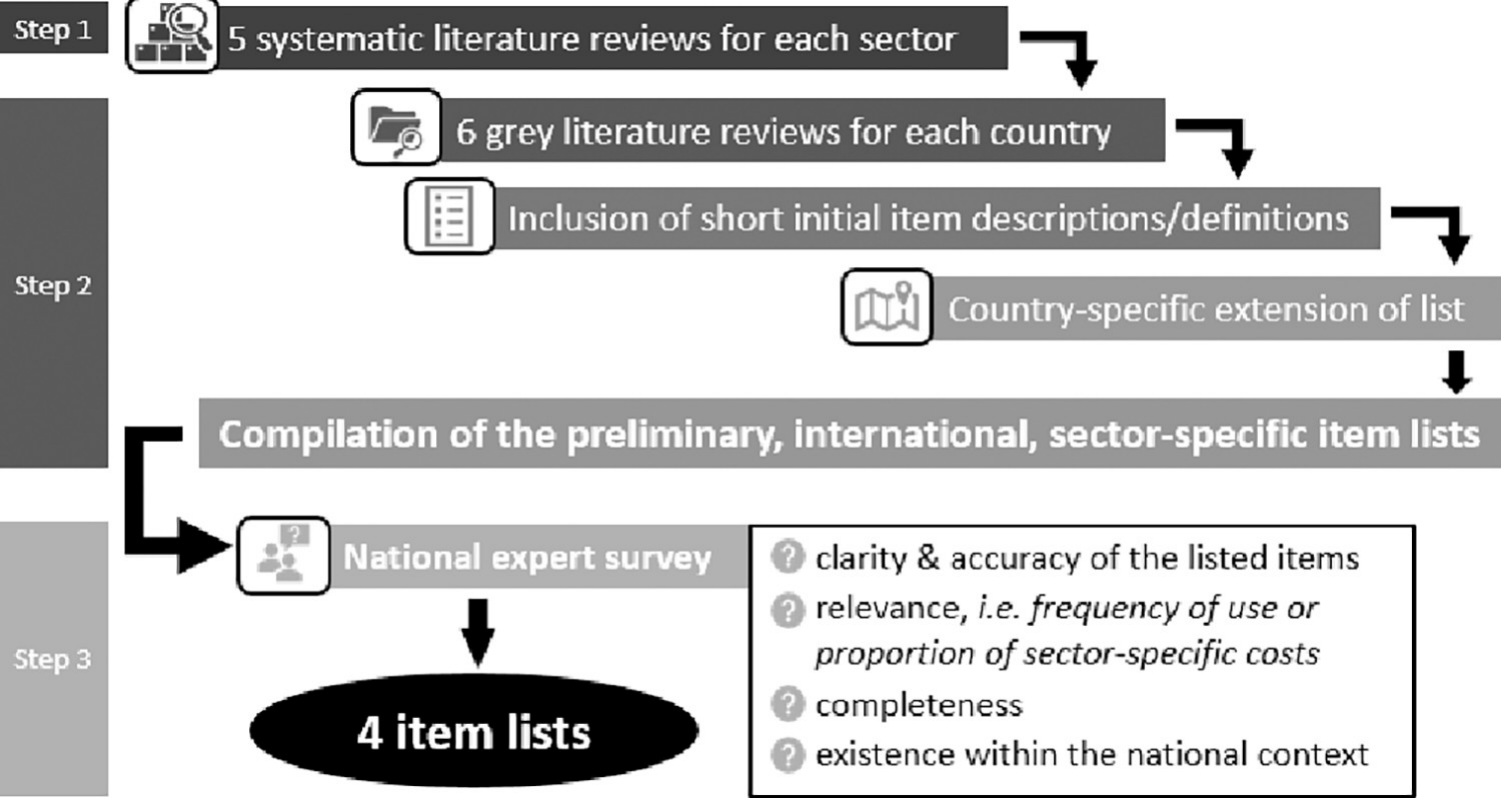
Iterative development process international sector-specific item lists.

An overview of the development process steps of the international sector-specific item lists is provided in Fig 1 and described in detail in the section below.

### Compilation of the preliminary, international, sector-specific item lists

The identification process commenced with systematic literature reviews to generate a preliminary, literature-based, international, mental health-related sector-specific service and resource use item lists. The searches were adapted to sector specific needs. For example, for the HCSC sectors the search focused on cost-of-illness studies and cost-effectiveness analyses. In contrast, the search for the CJ sector was not limited to a specific study design. Summaries of the main methodological characteristics of the conducted systematic literature searches and their necessary sector specific adaptions are presented in S1 Table. Full descriptions have been published elsewhere [33,34]. They included sector-specific, peer-reviewed publications and were conducted at sector level to serve as a scientific foundation. Further, country and sector-specific grey literature searches were carried out in all six selected countries to complement the preliminary, literature-based, sector-specific, mental-health related lists of services and other resource use items such as ICBs for the investigated sectors in Europe. Services were defined as “describ[ing] a combined and coordinated set of inputs (including structure, staff and organization) that can be provided to different user groups in a given sector (e.g. education) and under a common domain (e.g. child care), to improve the individual or population [health] status and/or functioning, or to attain a set of defined goals within a given sector.” [35].

The second step aimed at developing a first consolidated literature-based list of services and other resource use items such as ICBs in English for each included sector including short initial item descriptions/definitions (in MsExcel® 2013), hereinafter referred to as ‘preliminary item lists’. Definitions were taken from the original sources or based on internet search. Although the HCSC sectors are generally considered to be two separate sectors, services and experts from the two sectors are increasingly integrated around the needs of individuals, their carers and other family members. Therefore, it was decided to treat these two sectors in a combined manner and create one HCSC item list. The same applied to the item lists developed for PFI sectors.

For the HCSC sectors, the initial international literature-based list was further extended to accommodate for a special characteristic of the Austrian health care system. In Austria, outpatient specialist services (e.g. provided by a gynecologist) may be delivered both in the ambulatory care sector (i.e. in physician practices) and in hospital outpatient wards [13]. As unit costs for services provided inside or outside a hospital may be fairly different [36], further specifications of the items were necessary for the Austrian expert survey. Relevant outpatient items were thus separated into ‘outside the hospital, in physician practices’ versus ‘in hospital outpatient ward’.

Further in this step, any problems regarding the allocation of services to the specific sectors was tackled. In cases where one service potentially crossed multiple sectors, the service and its description were discussed in detail within the PECUNIA Consortium until consensus was reached between the team of researchers regarding the main sector of provision. This was based on the general rule developed and used in the PECUNIA costing concept that the sector specificity of a service should be determined by its provision and not its funding, since the latter one is often complex or blurry and cannot be allocated clearly to a single sector. In the case of some services, that the service had to be allocated to two different sectors and the service descriptions had to be adapted to enable a clear distinction of the scope of the activities distinguishing them (e.g. home assistance).

### National expert survey

The final step aimed at critically assessing these literature-based, sector-specific preliminary item lists by national experts as part of a multi-national expert survey. Based on the initial national grey literature search and additional systematic assessment of sector-specific websites and documents of Austrian institutions and stakeholders, potential experts were identified. Invited experts included stakeholders, decision-makers, people actively working in the field and researchers with sector-specific applied expertise. Initially identified experts were also asked to recommend additional experts (snowballing). A purposive sampling approach was adopted [37,38]. Austria being a federal state with fragmented competencies in the different sectors and regions, experts were recruited from both the national and regional levels to accommodate for potential regional variations. To allow for representative assessment, an equal number of experts from different stakeholder groups and a maximum of two experts per institution were invited. Two participating experts per sector were considered the minimum target. An overview of the experts considered for recruitment for the expert survey within the specific sectors are shown in S2 Table.

For the multi-national expert survey, survey instructions, standardized email templates and informed consent forms were created. Survey participants were invited to review each service list regarding the clarity of the listed items, relevance (frequency of use or proportion of sector-specific costs), completeness and existence within the national context. To this end, experts were firstly asked to comment on the naming and short descriptions of the listed items in terms of clarity and accuracy. Open-ended questions were included to also provide experts with the opportunity to comment on non-existing and missing items. Secondly, experts for the HCSC and PFI sectors were asked to assess the frequency of use based on the listed items for mental health patients in general as well as for the three exemplary disease categories (depression, schizophrenia and PTSD). Items with a use frequency of >10% were ‘prioritized’. In the surveys on the CJ and ED sectors, experts were additionally asked to rank the most important items (5 in the ED sector and 8 in the CJ sector) from an economic perspective based on frequency of occurrence/use and costliness from 1 (most important) to 5/8 (least important).

This section of the surveys consisted of closed-ended questions. The surveys including the item lists and short descriptions were generally provided in English and were designed to take between 30 and 60 minutes to complete. An overview of questions and answer options of the sector-specific expert surveys are provided in S3 Table.

An email invitation was sent out and experts were contacted where feasible mid-November 2018. These experts were provided with a written information leaflet (containing information on the study purpose, data security, etc.). No minors were included. Those experts who agreed to take part in the study were provided with the given sector-specific preliminary item list as well as a written informed consent form. In the consent form, experts could indicate whether they wanted their name and affiliation to be mentioned on the PECUNIA project website and/or in the acknowledgement section of any related publication, or if they preferred to stay anonymous altogether. The survey ran until the end of January 2019 with multiple reminders. When requested, experts were supported in the completion of the survey via phone. The expert survey study involved no patients or interventions, therefore, ethical approval by the Medical University of Vienna was not considered necessary. Survey results were analysed by calculating frequencies of the highly prioritized items, unclear and not existing items. Due to the limited number of responses and to assure data protection, expert survey data may be provided upon request from the corresponding author in aggregated form per sector.

## Results

### Preliminary, international, sector-specific item lists

**Fig 2:**
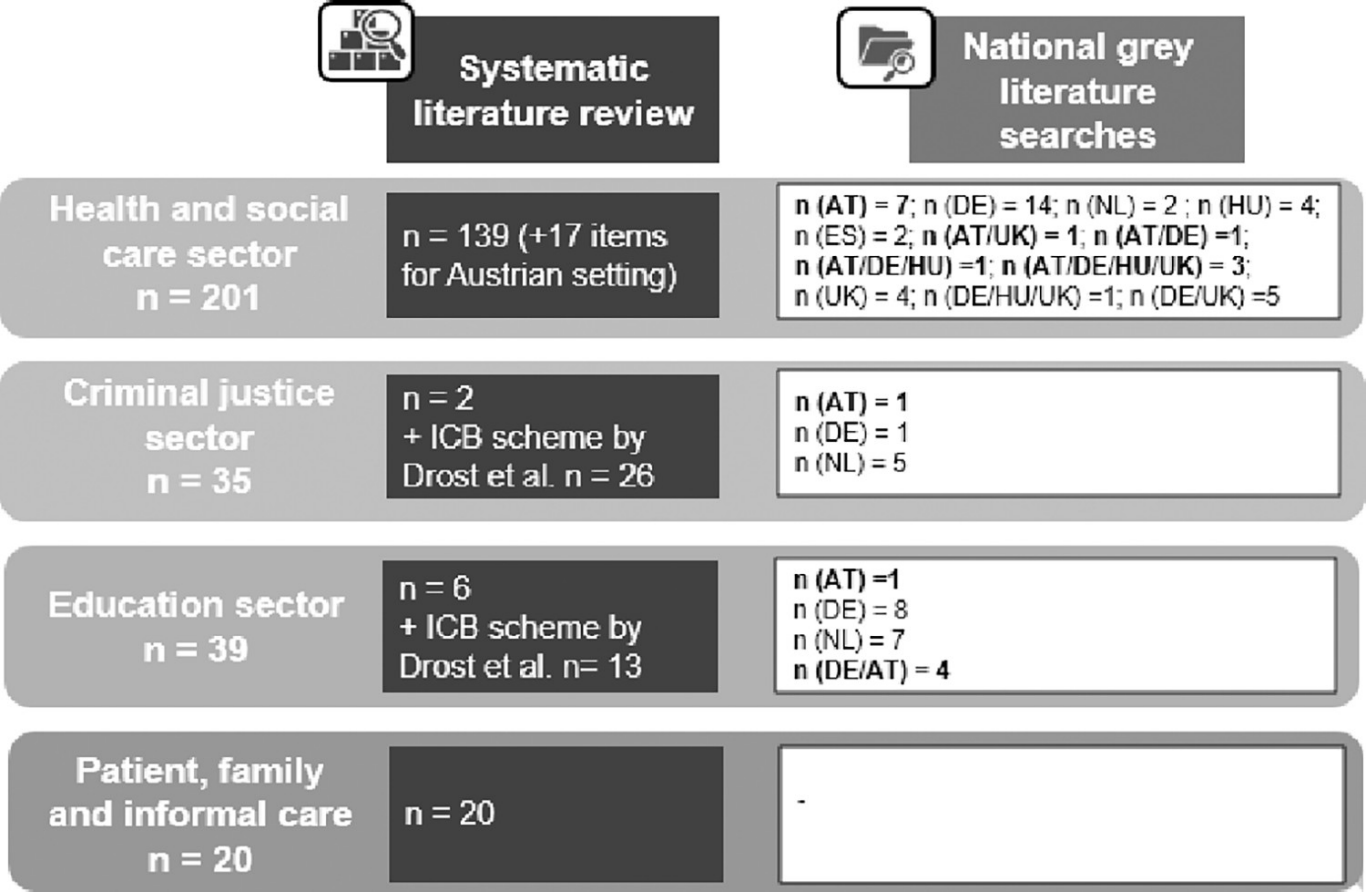
Sources of items in the preliminary, international sector-specific lists. Abbreviations: AT–Austria, DE–Germany, NL–the Netherlands, HU- Hungary, ES–Spain, UK–United Kingdom, ICB–inter-sectoral costs and benefits.

For the HCSC sectors, combining the accumulated items retrieved from the systematic literature reviews and the national grey literature searches resulted in a total of 201 items and revealed major difficulties in the differentiation of services and interventions suggestive of a considerable underlying typological problem based on item names. A total of 35 items were included in the list for the CJ sector and 39 items for the ED sector. For the list in the PFI sectors, 20 items were included based on the systematic literature review. An overview of the sources of the identified items in the preliminary, international sector-specific list is shown in Fig 2.

### National expert survey

#### Survey participants

Of the 83 Austrian experts who were initially invited to participate in the survey, 50 experts (60%) did not respond to the general email invitation, while 33 experts (40%) consented to take part in the study and were included in the survey. Among the experts who initially consented to take part, 15 (45%) did not return the survey. Only four experts reported their reasons for dropping out, including perceived data protection issues, inability to provide data-driven responses and language barriers. The other eleven experts were lost to follow-up. A total of 18 experts completed the sector-specific Austrian surveys, therefore, the pre-specified, minimally required responses by two experts per sector were achieved for all sectors (Table 1).

**Table 1:** Survey participants for Austria.

Sector	Invited to participate	Agreed to participate	Non-response	Actively withdrawn (after survey was sent)	Participated	Lost to follow-up
Healthcare	20	11	9	1	**5**	5
Social care	18	5	13	0	**2**	3
Criminal justice	7	2	5	0	**2**	0
Education	17	7	10	1	**4**	2
Patient	6	4	2	0	**3**	1
Family/Informal care	15	4	11	2	**2**	0
**TOTAL**	**83**	**33**	**50**	**4**	**18**	**11**

Note: Surveys for the healthcare and social care sectors were identical; surveys for the patient, family and informal care sectors were identical.

Among completers, 9 were female and 9 were male. The majority of these experts (n = 10) were based in Vienna (Salzburg: n = 3, Lower Austria: n = 2, Styria, Burgenland and Upper Austria: n = 1, respectively). Although no experts from the federal states Vorarlberg, Carinthia and Tyrol could be recruited, several of the participating experts were representatives of national institutions, and therefore they were able to provide national estimates in their survey responses. The expert survey included four representatives of patient organizations, three psychiatrists, two representatives of health insurance funds, four service providers, two policymakers and three representatives of federal state authorities.

#### Survey results

Out of a combined number of 295 items included in the preliminary item lists sent out to the experts, a total of 261 items and descriptions (88%) were considered clear by all experts. 43 items (15%) were considered not existing in Austria by at least one expert, and 110 items (37%) were prioritized in regards to their use frequency. A total of 13 additional items (4%) were suggested for addition to accommodate the Austria-specific features of the individual sectors. An overview of the results by sector is provided in the subsequent sections.

*Health and social care (HCSC) sectors*. Altogether, seven experts commented on 201 items in the preliminary HCSC sectors list. The items were grouped as inpatient ((non-)mental health hospital unit, medical/laboratory procedures), outpatient (outpatient (non-) mental health-specific physician, outpatient non physician (nursing services, alternative services/institutions, pharmacy)), cross-categorial (including both, inpatient and outpatient services) services (rehabilitative, psychiatric, therapeutic and diagnostic procedures), and non-medical costs for (social) support, living support and vocational support. 77 items (38.3%) had an estimated use frequency >10% of persons with mental diseases and were prioritized (see items in bold). Details on the perceived item clarity (twenty-one unclear items (10,4%)) and existence (twenty-six non-existent items (12,9%)), as well as the six suggested additional items can be seen from Table 2.

**Table 2:** Characteristics of items in the health and social care (HCSC) sectors list, Austrian survey.

Nr.	Items	Item clear	Non-existing item
Inpatient			
General hospital			
Academic hospital			
Non-mental health hospital unit			
1	Polyclinic^a^	5/7	1/4
2	Surgical unit^a^	7/7	0/4
3	**Neurological unit** ^a^	7/7	0/4
4	Hematology/oncology unit^a^	7/7	0/4
5	Intensive care unit/critical care unit^a^	7/7	0/4
6	Coma care unit^b^^c^	7/7	0/3
7	Sleep clinic^a^	6/7	0/4
8	Emergency room^a^	5/7	0/4
9	First aid station^a^	5/7	0/4
10	**Ambulance ride** ^a^	7/7	0/4
11	Paramedic^a^	7/7	0/4
Mental-health specific hospital unit			
12	**Psychiatric daycare unit** ^a^	6/7	0/4
13	Psychiatric ward^a^	6/7	0/4
14	**Acute psychiatric ward** ^a^	7/7	0/4
15	Long-term ward^a^	7/7	1/4
16	**(Psycho-)geriatric ward** ^a^	7/7	0/4
**17**	Soteria-ward^b^	7/7	1/4
**18**	Rehabilitation facility^a^	5/7	0/4
**19**	Psychiatric intensive care unit (PICU)^a^	6/7	0/4
*Medical/laboratory procedures*			
20	MRI (brain, lower extremities, upper extremities)^a^	7/7	0/4
21	CT scan (brain, lower extremities, upper extremities)^a^	7/7	0/4
22	Ultrasound (skull, lower extremities, upper extremities)^a^	7/7	0/4
23	**X-ray (chest)** ^a^	7/7	0/4
24	**Electrocardiogram (ECG)** ^a^	7/7	0/4
25	Single Photon Emission Computed Tomography (SPECT)^a^	7/7	0/4
26	Neuropsychological examination^a^	7/7	0/4
27	Lumbar tap^a^	7/7	0/4
28	Retinitis pigmentosa test (RP test)^a^	7/7	0/4
29	Blood products (erythrocytes, platelets pooled in plasma, platelets)^b^	7/7	0/4
30	**Blood tests** ^a^	7/7	0/4
31	HbA1C test^a^	7/7	0/4
32	**TSH test** ^a^	7/7	0/4
33	**Creatinine test/clearance** ^a^	7/7	0/4
34	**Liver function test** ^a^	7/7	0/4
35	Antibody test^a^	7/7	0/4
36	Syphilis test^a^	7/7	0/4
37	APOE4-test^a^	7/7	0/4
38	**Urine tests** ^a^	7/7	0/4
39	Kidney function^a^	7/7	0/4
Outpatient			
Outpatient physician			
Mental-health specific physician			
40	**Psychotherapist in hospital outpatient ward** ^a^	5/7	0/4
41	**Psychotherapist outside the hospital, in physician practice** ^c^	5/7	1/4
42	**Psychologist in hospital outpatient ward** ^a^	7/7	0/4
43	**Psychologist outside the hospital, in physician practice** ^c^	6/7	1/4
44	**Neurologist/psychiatrist in hospital outpatient ward** ^a^	6/7	0/4
45	**Neurologist/psychiatrist outside the hospital, in physician practice** ^c^	6/7	0/4
Non-mental-health specific physician			
46	**General practitioner** ^a^	7/7	0/4
47	**Standard consultation** ^a^	7/7	0/4
48	**Physical health monitoring** ^b^	7/7	0/4
49	Home visit^a^	6/6	0/4
50	Telephone contact^a^	7/7	0/4
51	**Practice supporter** ^a^	7/7	0/4
52	**GP assistant** ^a^	7/7	3/4
53	Radiologist in hospital outpatient ward^a^	7/7	0/4
54	**Radiologist outside the hospital, in physician practice** ^c^	7/7	0/4
55	Urologist in hospital outpatient ward^a^	7/7	0/4
56	Urologist outside the hospital, in physician practice^c^	7/7	0/4
57	Gynecologist in hospital outpatient ward^a^	6/7	0/4
58	**Gynecologist outside the hospital, in physician practice** ^c^	6/7	0/4
59	Orthopedist in hospital outpatient ward^a^	7/7	0/4
60	**Orthopedist outside the hospital, in physician practice** ^c^	7/7	0/4
61	Dermatologist in hospital outpatient ward^a^	7/7	0/4
62	Dermatologist outside the hospital, in physician practice^c^	7/7	0/4
63	Otolaryngologist in hospital outpatient ward^a^	7/7	0/4
64	Otolaryngologist outside the hospital, in physician practice^c^	7/7	0/4
65	Dentist in hospital outpatient ward^a^	7/7	1/4
66	**Dentist outside the hospital, in physician practice** ^c^	7/7	0/4
67	Cardiologist in hospital outpatient ward^a^	7/7	0/4
68	Cardiologist outside the hospital, in physician practice^c^	7/7	0/4
69	Ophthalmologist in hospital outpatient ward^a^	7/7	0/4
70	**Ophthalmologist outside the hospital, in physician practice** ^c^	7/7	0/4
71	Internist in hospital outpatient ward^a^	7/7	0/4
72	Internist outside the hospital, in physician practice^c^	7/7	0/4
73	Chiropodist in hospital outpatient ward^a^	7/7	1/4
74	Chiropodist outside the hospital, in physician practice^c^	7/7	1/4
75	Geriatrician in hospital outpatient ward^a^	7/7	0/4
76	Geriatrician outside the hospital, in physician practice^c^	7/7	0/4
77	Surgeon in hospital outpatient ward^a^	7/7	0/4
78	Surgeon outside the hospital, in physician practice^c^	7/7	0/4
79	Oncologist in hospital outpatient ward^a^	7/7	0/4
80	Oncologist outside the hospital, in physician practice^c^	7/7	0/4
Outpatient non-physician			
Nursing services			
81	District nurse^a^	7/7	2/4
82	Community psychiatric nurse^a^	7/7	2/4
83	Psychiatric nurse^a^	7/7	2/4
84	GP nurse/practice nurse^a^	7/7	2/4
85	Consultative psychiatric nurse^a^	7/7	2/4
86	Registered nurse^a^	7/7	2/4
87	**Psychiatric home nursing service** ^a^	7/7	2/4
88	Anticoagulant service^a^	7/7	2/4
Alternative services/institutions			
89	**Counselling (non-physician)** ^a^	7/7	0/4
90	**Family counselling** ^a^	7/7	0/4
91	**Marriage/couples counselling** ^a^	7/7	0/4
92	**Group counselling** ^a^	7/7	0/4
93	Addiction counselling^b^	7/7	0/4
94	**Optician** ^a^ ** **	7/7	0/4
95	Dietician^a^	7/7	0/4
96	Hypnotherapy^b^	7/7	0/4
97	**Occupational therapy** ^a^	7/7	0/4
98	Sociotherapy^a^	7/7	0/4
99	**Physical therapy/manual therapy** ^a^	7/7	0/4
100	**Psychoeducation** ^b^	7/7	0/4
101	Speech therapy^a^	7/7	0/4
102	Dance therapy^b^	7/7	0/4
103	Movement therapy^b^	7/7	0/4
104	Art therapy^a^	7/7	0/4
105	Music therapy^a^	7/7	0/4
106	Theatre therapy^a^	6/7	1/3
107	Relaxation therapy^a^	7/7	0/4
108	Anthroposophical therapy^a^	6/7	0/3
109	**Interdisciplinary pedagogical projects** ^a^	7/7	0/4
110	**Social-skills-training** ^b^	7/7	0/4
111	Biofeedback^b^	7/7	0/4
112	Exercise therapy^b^	7/7	0/4
113	Therapeutic (rock) climbing^b^	7/7	0/4
114	Therapeutic running^b^	6/7	0/3
115	Therapeutic yoga^a^	7/7	0/4
116	**Animal therapy** ^b^	7/7	0/4
117	**Dog-therapy** ^b^	7/7	0/4
118	**Hippotherapy** ^a^	7/7	0/4
119	Service animal/emotional support animal^a^	7/7	0/4
120	Alternative practitioner^a^	7/7	0/4
121	Homeopathic practitioner^a^	7/7	0/4
122	Naturopath^a^	7/7	0/4
123	Acupuncturist^a^	7/7	0/4
124	Massage therapist^a^	7/7	0/4
Pharmacy			
125	**Community pharmacist** ^a^	7/7	0/4
126	Pharmacist dispensing cost^a^	6/7	1/3
127	**Drugs/medication** ^a^	7/7	0/4
128	**Over-the-counter drugs** ^a^	7/7	0/4
129	**Prescription drugs** ^a^	7/7	0/4
Cross-categorial (inpatient/outpatient)			
Rehabilitative procedures			
130	Addiction rehabilitation^b^	7/7	0/4
131	**Drug rehabilitation therapy** ^a^	7/7	0/4
132	**Rehabilitation aftercare** ^a^	7/7	1/4
Psychiatric procedures			
133	Electroconvulsive therapy^b^	7/7	0/4
134	Repetitive Transcranial Magnetic Stimulation (rTMS)^b^	7/7	0/4
135	Vagus-nerve-stimulation^b^	7/7	0/4
136	**Eye Movement Desensitization and Reprocessing (EMDR)** ^b^	7/7	0/4
137	Cognitive rehabilitation^b^	7/7	0/4
138	**Sleep deprivation therapy** ^b^	7/7	0/4
139	**Light therapy** ^a^	7/7	0/4
Therapeutic procedures			
140	**(Cognitive) behavioral therapy** ^a^	7/7	0/4
141	Computerized cognitive behavioral therapy^a^	6/7	0/3
142	Psychodynamic therapy^b^	7/7	0/4
143	**(Psychodynamic) interpersonal therapy** ^b^	7/7	0/4
144	**Problem solving therapy** ^b^	7/7	0/4
145	Psychoanalysis^a^	7/7	0/4
146	Dialectic behavioral therapy^a^	7/7	0/4
147	**Systemic psychotherapy** ^b^	7/7	0/4
148	**Supportive psychotherapy** ^a^	7/7	0/4
149	**Non-directive psychotherapy** ^b^	7/7	0/4
150	Low-intensity psychosocial interventions^a^	7/7	0/4
151	**High -intensity psychosocial interventions** ^a^	7/7	0/4
152	Watchful waiting^b^	7/7	0/4
153	Early intervention^a^	7/7	0/4
154	**One-on-one therapy** ^a^	7/7	0/4
155	**Group therapy** ^a^	7/7	0/4
156	Family therapy^a^	7/7	0/4
157	Milieu therapy^b^	7/7	0/4
*Diagnostic procedures*			
158	**Personality tests** ^b^	7/7	0/4
159	**Intelligence tests** ^a^	7/7	0/4
Non-medical costs			
(Social) support			
160	**Home assistance** ^a^	7/7	0/4
161	Paid home help/home aid^a^	7/7	0/4
162	Legal carer/guardian^b^	7/7	0/4
163	**Elderly mentally impaired care/(psycho-)geriatric home care** ^a^	7/7	0/4
164	Meals-on-wheels/ food delivery^a^	7/7	0/4
165	Social assistance^a^	7/7	0/4
166	**Social worker** ^a^	7/7	0/4
167	**Mental health worker** ^a^	7/7	0/4
168	**(Intensive) case manager** ^a^	7/7	0/4
169	**Crisis resolution worker** ^a^	7/7	0/4
170	**Drug and alcohol worker** ^a^	7/7	0/4
171	Escort/accompanied leave^a^	7/7	1/4
172	Psychosocial crisis center^a^	7/7	1/4
173	Counselling center/ advice center^a^	7/7	0/4
174	Drop-in center^a^	7/7	0/4
175	Meeting facility^b^	7/7	0/4
176	**Self-help groups** ^a^	7/7	0/4
177	Support helplines^a^	7/7	0/4
178	**Support groups** ^a^	7/7	0/4
179	Parenting group programs^a^	7/7	0/4
180	Hyperactivity support^a^	7/7	1/4
181	Community services/support^a^	7/7	1/4
182	Internet-based interventions^a^	6/7	0/3
Living support			
183	Assisted living facility^a^	7/7	0/4
184	Assistant tenant group^b^	6/6	1/4
185	**Psychiatric residential home** ^b^	7/7	0/4
186	Social care facility^b^	7/7	0/4
187	Day-care^a^	7/7	0/4
188	Long-term care^b^	7/7	0/4
189	Homeless shelter/women’s shelter^b^	7/7	0/4
Vocational support			
190	**Company physician** ^a^	7/7	0/4
191	**Company nurse** ^a^	7/7	1/4
192	**Company psychologist/counsellor** ^a^	7/7	0/4
193	**Company social worker** ^a^	7/7	0/4
194	Protected/sheltered workshop^a^	7/7	0/4
195	Integration workplace^b^	7/7	0/4
196	Individual vocational qualification^b^	7/7	0/4
197	Professional training^b^	7/7	0/4
198	Integration services^b^	7/7	0/4
199	Proficiency testing^b^	7/7	0/4
200	**Supported employment programs** ^b^	7/7	0/4
201	Pre-vocational training^b^	7/7	0/4
**Additional items suggested by experts**			
	Psychiatric hospital (a hospital exclusively for psychiatric patients)		
	Specialized hospital (any specialty) (a hospital exclusively for a specific group of patients (e.g. orthopedic hospital))		
	Psychiatric mobile services		
	Medical doctor providing treatment during night or during weekend		
	Psychiatric services providing assertive outreach		
	Caregivers self-help groups		

a–Systematic literature review

b–grey literature review

^c^–added for the Austrian setting.

^$^ Items in bold indicate that they were prioritized (used annually by >10% by persons with mental diseases) by at least one of the experts.

*Criminal justice (CJ) sector*. Two experts reviewed the 35 items in the preliminary CJ sector list. The items were grouped as costs incurred as a consequence of crime (including offences against the person or property; psychological, material or other crime consequences) and costs incurred in response to crime (including law enforcement, victim/witness support and other). Nine items (25.7%, six items in the section ‘costs incurred as a consequence of crime’, three items in the section ‘costs incurred in response to crime’) were ranked among the most important items from an economic perspective by at least one of the experts (see items in bold). The three considered unclear items (8.6%) and three non-existent items in Austria (8.6%) can be seen in Table 3. No new items for suggested for the CJ sector list.

**Table 3:** Characteristics of items in the criminal justice sector (CJ) sector list, Austrian survey.

Nr.	Items	Item clear	Non-existing item
Costs incurred as a consequence of crime			
Offences against the person			
1	**Domestic violence**^a^^,^ ^$^	2/2	0/2
2	**Unlawful threats** ^a^	2/2	0/2
3	**Assaults (offences)** ^a^	2/2	0/2
4	**Violence towards officials** ^a^	2/2	0/2
5	Drunk driving (accidents)^a^	2/2	0/2
6	Child maltreatment* ^a^	2/2	0/2
7	**Sexual assaults** ^a^	2/2	0/2
8	Homicide^a^	2/2	0/2
Offence against property			
9	**Vandalism** ^a^	2/2	0/2
10	Theft^a^	2/2	0/2
Crime consequences psychological			
11	Pain and suffering of victims^a^	2/2	0/2
12	Pain and suffering of others^a^	1/2	1/2
13	Long term consequences of victimizations^a^	2/2	0/2
14	Victimization of offenders while incarcerated^a^	2/2	0/2
Crime consequences material			
15	Loss of property of victims^a^	2/2	0/2
16	Loss of property of others^a^	2/2	0/2
Crime consequences other			
17	Lost work/productivity of victims^a^	2/2	0/2
18	Lost work/productivity of offender^a^	2/2	1/2
19	Illegal untaxed income by primary person^a^	2/2	0/2
20	Lost freedom to the offender^c^	1/1	0/2
Costs incurred in response to crime			
Law enforcement			
21	**Police services/interventions** ^a^	2/2	0/2
22	Prison expenditures^b^	2/2	0/2
23	Judicial expenses^a^^,^**	2/2	0/2
24	Institutionalization/incarceration of juveniles or adults^a^	2/2	0/2
25	Housing stock lost^a^	2/2	0/2
26	Services for children of incarcerated^a^	2/2	0/2
27	Probation^a^	1/2	0/2
28	Parole *(incl*. *electronic monitoring* ^a^	2/2	0/2
29	**Fire and rescue services** ^c^	2/2	0/2
30	Forensic (psychiatric) services *(including aftercare)*^b^	2/2	0/2
31	Costs of correctional institutions^b^	2/2	0/2
Victim/witness support			
32	Victim/witness protection^c^	2/2	0/2
33	Victim compensation^c^	2/2	0/2
Other			
34	**Programs regarding the improvement of mental health of the offender** ^c^	2/2	0/2
35	Decreased chance of (committing a) crime as a consequence/effect of mental health programs/interventions^c^	0/2	1/2

a–Classification scheme by Drost et al. (2013) [15]

b–systematic literature review

c–grey literature search.

^$^ Items in bold indicate that they were prioritized (ranked among the top 5 (costs incurred as a consequence of crime) and top 3 (costs incurred in response to crime) most important items from an economic perspective) by at least one of the experts.

* Including abuse and neglect.

** Including lawsuits, custody, prosecution, fines and transactions, tort claims, offender costs, legal defense, criminal sanctions, jury services, mediation and trustee.

*Education (ED) sector*. Four experts commented on the 39 items in the preliminary list for the ED sector, which were grouped into intangible and tangible ICBs. Tangible ICBs encompassed services within the educational sector aimed at students with mental diseases (i.e. special education). Intangible ICBs referred to the consequences of mental health diseases experienced by students that could have an influence on the educational sector (i.e. cognitive deficits). The idea behind the chosen classification reflects the aspects of the impact of mental health interventions on either costs (or benefits) or outcomes. Therefore, tangible ICBs are most likely to lead to costs (or benefits) in the educational sector, while intangible ICBs will probably affect the quality of life. Eleven items (28%) were ranked among the most important items from an economic perspective by at least one of the experts (see items in bold). Table 4 shows further details on the four items (10%) that were considered to be not clear, the five items (13%) reported to be not existent in Austria and the five additional items (13%) suggested to be added by the experts.

**Table 4:** Characteristics of items in the education (ED) sector list, Austrian survey.

Nr.	Items	Item clear	Non-existing item
**Tangible inter-sectoral costs and benefits (ICBs)**			
1	**Special education service** ^**a**^^,^ ^$^	4/4	0/4
2	**Learning therapy** ^c^^,^ ^*^	3/4	0/4
3	Home education^a^	4/4	1/4
4	**School-based health promotion interventions** ^b^	4/4	0/4
5	**Liaison teacher** ^c^	4/4	0/4
6	**Compensation for disadvantages** ^c^	4/4	0/4
7	**Learning therapy**^c^^,^ ^**^	1/3	0/2
8	Special needs diagnostics^c^	3/4	0/4
9	Counseling of legal guardians^c^	4/4	0/4
10	Student counselling^c^	3/3	0/3
11	Temporary study group^c^	4/4	0/4
12	Social and educational therapy boarding school^c^	4/4	0/4
13	Night school^c^	4/4	3/4
14	Attendance officer^c^	4/4	2/3
15	Student transport to special education facility^c^	4/4	0/4
16	Student-related financing^c^	4/4	0/4
**Intangible inter-sectoral costs and benefits (ICBs)**			
17	**Change in school readiness** ^a^	3/4	0/3
18	Problems with school entry^a^	4/4	0/4
19	Learning disabilities^a^	4/4	0/4
20	Cognitive deficits^a^	4/4	0/4
21	Low school adaptation/competence^a^	4/4	0/4
22	Low school participation/engagement^a^	4/4	0/4
23	Low school attainment/productivity/performance^a^	4/4	0/4
24	Grade retention^a^	4/4	1/4
25	Disrupted school experience^a^	4/4	0/4
26	**Teacher-student conflicts** ^a^	4/4	0/4
27	**School dropout/pre-mature leave** ^a^	4/4	0/4
28	**Indirect effect of premature school leave/drop-out** ^c^	4/4	0/4
29	**(Social) reintegration** ^c^	4/4	0/4
30	Inclusion^c^	4/4	0/4
31	Refusal of admission^c^	4/4	0/3
32	Change in educational level^c^	4/4	0/4
33	Exemption from compulsory education^c^	3/3	0/3
34	Talent development^b^	4/4	0/4
35	Discrimination^b^	4/4	0/4
36	Peer relations^b^	4/4	0/4
37	Suspension^c^	4/4	1/4
38	Negative feelings about school^**b**^	4/4	0/4
39	Classroom behaviour^**b**^	4/4	0/4
**Additional items suggested by experts**			
	Support staff, support conferences and support material		
	School assistants for everyday needs		
	Additional lessons		
	Professional therapy (e.g. for children with exposure to violence)		
	Impact of parents and relatives (the influence of people who are also part of the school system but not working for the system)		

a–Classification scheme by Drost et al. (2013) [15]

b–systematic literature review

c–grey literature review.

^$^ Items in bold indicate that they were prioritized (ranked among the 3 (tangible items) and 5 (intangible items) most important items from economic perspective (based on frequency of occurrence and costliness)) by at least one of the experts.

* Includes e.g. the diagnosis of learning disability with regards to reading, writing, calculating or weak concentration; individual therapy and treatment plans; counselling and thereby fostering individual strengths, skills and talents.

** Special pedagogical therapy to support children with learning disabilities.

*Patient*, *family and informal care (PFI) sectors*. Five experts reviewed the preliminary item list for the PFI sectors in regard to their clarity and non-existing/additional items for Austria. The items were grouped into four categories: informal care, services, goods and others. Fourteen items were ranked among the most important items from an economic perspective by at least one of the experts. All of the items within the ‘informal care’ section were clear, whereas six (30%) items in the ‘services’, ‘goods’ and ‘other’ sections were regarded as unclear by one expert. Eight items (40%) were regarded to be non-existent in Austria by at least one of the five experts. Two additional items were suggested to be added to the list (Table 5).

**Table 5:** Characteristics of items in the patient, family and informal care (PFI) sectors list, Austrian survey.

Nr.	Items	Item clear	Non-existing item
**Informal care**			
1	**Domestic assistance (household activities)**^a^^,^ ^$^	5/5	0/5
2	**Personal care** ^a^	5/5	0/5
3	**Practical support** ^a^	5/5	0/5
4	**Supervision** ^a^	5/5	0/5
**Services**			
5	**Organized volunteer care** ^a^	5/5	0/5
6	**Paid non-professional personal care** ^a^	4/5	0/5
7	**Paid domestic assistance** ^a^	5/5	0/5
8	Paid babysitting (while the parents are temporarily away due to the illness)^a^	5/5	1/5
9	**Alternative forms of psychiatric rehabilitation** ^a^	5/5	0/5
10	**Non-professional treatment** ^a^	5/5	1/5
11	**Public or private transportation** ^a^	5/5	0/5
12	**Phone calls** ^a^	5/5	0/5
13	Carer conference or training attendees^a^	4/5	0/5
14	Home adaptation^a^	5/5	2/5
15	Accommodation cost of caregiver^a^	4/5	1/5
**Goods**			
16	Durable goods/specialist equipment^a^	5/5	2/5
17	Consumable goods^a^	4/5	2/5
**Other**			
18	**Disease-related loss of net income** ^a^	4/5	1/5
19	**Changes to patient living accommodation** ^a^	4/5	0/5
20	**Costs of cancelling holidays** ^a^	5/5	1/5
**Additional items suggested by experts**			
	Case manager		
	Relations discounting		

a–Systematic literature review.

^$^ Items in bold indicate that they were prioritized (used annually by at least >10% by persons with mental diseases) by at least one of the experts.

## Discussion

For the comprehensive and internationally comparable assessment of the cost impact of mental diseases from a societal perspective, an overview of the potentially relevant services and resource use is a key prerequisite. This manuscript summarizes a first attempt by detailing the iterative development process of four item lists based on a cross-country and cross-sectoral harmonization strategy as part of the ongoing European PECUNIA project with a focus on Austria. The item lists were designed to include all services and other resource use items including ICBs that are relevant for assessing the consequences of mental health-related interventions in the Austrian HCSC, ED, CJ and PFI sectors. The need for such a step has already been established as part of the earlier European MHEEN study not only for therapeutical interventions but also for mental health promotion and prevention [39].

Preliminary, international, sector-specific item lists were subjected to an expert review as part of a multi-national survey with regards to their clarity, descriptions, frequency of use and completeness. Out of a combined number of 295 items included in all lists, a total of 261 items and descriptions (88%) were considered clear by all experts, 42 items (14%) were considered non-existent in Austria by at least one expert, while a total of 13 additional items (4%) were suggested to be added to accommodate for Austria-specific features of the individual sectors.

While the item list for the CJ sector was considered complete, for the other three sectors additional items were suggested to be relevant for Austria. In terms of the six items that were considered as currently missing from the HCSC sectors list, two items referred to the inpatient sector (psychiatric hospital, specialized hospital), and three items to the outpatient sector. The suggested item ‘caregiver self-help groups’ was indeed already covered in the list for the PFI sectors. The new items that were deemed relevant for the Austrian ED sector included additional staff and additional lessons. One additional item (professional therapy for children with exposure to violence) could be considered as covered also in the list of the HCSC sectors. Another suggestion (impact on parents and relatives) can be categorized as intangible item. For the PFI sectors, the two additionally suggested items include one service (case manager) and one other item (relations discounting).

This first identification step in the PECUNIA project revealed several points potentially specific to the Austrian context. Firstly, it was necessary to slightly adapt the international item list for the Austrian HCSC sectors already prior to the expert survey. In Austria, some outpatient specialist services may not only be delivered in hospital outpatient wards as captured within the international item list but also in the ambulatory care sector (i.e. in physician practices). Such a specification was therefore added where relevant, resulting in the ex-ante inclusions of a total of 17 additional Austria-specific items. Thirdly, completion of the questions related to the economic relevance of the listed items in the Austrian HCSC sectors was complicated by the lack of administrative data [40]. As pointed out by participating experts, data for the Austrian inpatient sector data is available in terms of diagnoses but not with regards to actual service use. Hence, a data-driven completion of this part of the survey was deemed impossible. At the same time, it was pointed out that data availability is a more general problem, as also for the outpatient sector reliable estimates of the economic relevance would be difficult to provide due to the absence of publicly available data. This might also explain why despite extensive efforts to identify and recruit experts for the surveys in the different sectors, the participation rate amongst those experts who had initially indicated to be willing to participate in the survey remained low. In addition, in four cases (57%) information on the frequency of the service use or economic relevance of the listed items for mental health diseases was missing in the returned surveys. This especially applies to the surveys in the HCSC sectors and may be due to the long list of items (n = 201) in comparison to the other sectors. For the PFI sectors, the question on the relevance of the listed items was also considered challenging, albeit for a different reason. Austrian experts specifically pointed out the gap between the actual use of the listed items as captured in the survey question and the potential users’ needs for them, which seems to be considerable in the PFI sectors. Another reason may lie in the perceived variation in disease severity even within one disease area (e.g. mild depression versus severe depression) and perceived variations in service use during disease episodes within one year that hamper such general assessments, as reported back by two experts.

All sector-specific surveys and item lists were developed in English as part of the cross-country and cross-sector harmonization approach. Generally, experts did not seem to have language issues. However, significant language barriers occurred in the PFI sectors, where experts preferred to fill out a translated German-language survey. This is likely to be related to differences in the professional profile of these experts. It may also explain the overall difficulties in recruiting experts for the PFI sectors specifically in addition to the still very limited practice of involving patients’ and carers’ views in research in Austria.

Further, a considerable variation in prioritization of items in regards to the individual economic importance by the experts was observed. This might be a reflection of national vs. regional variations in HCSC services.

The study also revealed important general points relevant to multi-sectoral, multi-national costing studies within the European context. Firstly, despite generally being considered as two separate sectors, one (combined) list of items was developed for the HCSC sectors. This was necessary due to the high level of variations that exist between countries in terms of the funding and provision arrangements whereby the two areas of service use may not be clearly distinguishable and separation of the item lists would be extremely challenging and not feasible with European-level comparability. A similar issue emerged in the case of the PFI sectors. Secondly, we experienced major difficulties in the differentiation of services and interventions based on item names suggestive of considerable underlying typological problems both in the literature and by experts. Thirdly, there was a great imbalance in the number of items and in the feasibility of finding sector-specific experts between the HCSC and the CJ and ED sectors confirming the need for additional mapping of the latter sectors for health-related services.

### Strengths and limitations

To the best of our knowledge, this is the first attempt both internationally and nationally to summarize mental health-related resource use comprehensively across several sectors, across six European countries based on iterative, harmonized methods including literature reviews as well as expert surveys. At the same time, the developed preliminary item lists and their assessment need to be interpreted in light of potential limitations. Firstly, the identification of experts for all considered sectors was challenging in all countries, although, through the identification of relevant stakeholders based on the national literature search and the application of snowballing techniques in expert recruitment, a sufficient pool of sector-specific experts could be determined in Austria. Secondly, in light of the above explored low sector-specific response rates the voluntary participation might have led to a selection bias. Further, the observed variation in response quality required assumptions regarding missing responses, therefore, future research may be necessary to validate and confirm the experts’ prioritization and completeness of the developed lists for Austria. To achieve this, especially in the CJ and ED sectors, additional mapping work of service availability would be needed. Thirdly, for all sector-specific surveys, experts were recruited from the national, regional and local level to accommodate for regional differences in service provision within Austria. Inevitably, variations in responses may therefore also be due to actual regional variations, which was not further elaborated in the present study. This especially affects the items classified as ‘non-existing’ in Austria. While these items were chosen from the list by at least one expert, conversely all other experts implicitly confirmed the existence of these items on the basis of their regional expertise. It is possible that confounding factors were not mitigated sufficiently considering that expert opinion-based studies are often based on personal judgements [41]. In future studies, a Delphi approach could be considered to tackle this issue and enhance decision-making in a systematic manner [42–44].

## Conclusion

The identified country-specific variations and general typological bias and their potential contributions to service and other resource use cost variations across countries and sectors call for further systematic investigation. In the next steps, PECUNIA will develop internationally harmonized and comparable definitions of the listed items and a new conceptual multi-sectoral costing framework. In addition, the developed lists will need to be consolidated across the six selected European countries and further prioritized for the development of a patient-reported RUM instrument and consequent reference unit cost valuation.

## Supporting information

S1 Table Main methodological characteristics of conducted literature searches. (DOCX)

S2 Table Overview of the experts considered for recruitment for the expert survey within the specific sectors. (DOCX)

S3 Table Design of the sector-specific expert surveys. (DOCX)
