# Supplementary material for: Establishing a comprehensive list of mental health-related services and resource use items in Austria: A national-level, cross-sectoral country report for the PECUNIA project
Source: PLoS One. 2022 Jan 21;17(1):e0262091. doi: 10.1371/journal.pone.0262091 (PMC8782519; doi:10.1371/journal.pone.0262091)
Supplement: S1 Table — (DOCX) [file pone.0262091.s001.docx]

**Appendix 1**

**S1 Table: Main methodological characteristics of conducted literature searches**

|  | **Health and social care sector** | **Criminal justice sector and education sector*** | **Patient, family and informal care sectors** |
| --- | --- | --- | --- |
| **Data source** | PubMed-Medline, PsycInfo, NHS EED, EconLit, Embase and DIRUM | PubMed | PubMed |
| **Search terms/ search string** | **#1 Mental Health component:**  [“Mental Disorders” [Mesh] OR mental OR dement* OR Alzheimer OR “cognitive impairment” OR addiction OR "substance abuse" OR "dependency" OR psychotic* OR schizophreni* OR delusional OR manic OR affective OR bipolar OR depressi* OR "depressive disorder" OR anxiety OR panic OR phobia OR "posttraumatic stress disorder*" OR "somatoform disorder" OR somatiz* OR somatis* OR "conversion disorder" OR “dissociative disorder” OR multisomatoform* OR neurotic* OR "medically unexplained symptoms" OR "medically unexplained illness" OR psychogen* OR nonorgan* OR (psychosom* syndrome) OR "functional somatic syndrome" OR "functional syndrome" OR hypochondri* OR "illness phobia" OR "health anxiety" OR "body dysmorphic disorder" OR dysmorphophobiaOR "anorexia nervosa" OR "bulimia nervosa" OR binge* OR purging OR "eating disorder*" OR "personality disorder*" OR schizoid OR schizot* OR "behavioural disorder*" OR "behavioral disorder*” OR "obsessive-compulsive disorder*" OR “impulse disorder*” OR “gender identity disorder*” OR “intellectual disabilit*” OR “developmental disorder*” OR “attention-deficit disorder*” OR “attention-deficit hyperactivity disorder*”] / “Mental Disorders” [Mesh]  **# 2 Economic component:**  [“cost-effectiveness” OR “cost-utility” OR “cost-benefit” OR "economic evaluation" OR cost]  **# 3 Country component:**  [german* OR austria* OR hungar* OR Netherlands OR dutch OR spain OR spanish OR UK] | “inter-sectoral” OR “indirect” OR “spillover” OR “external” OR “societal” OR “education” OR “criminal justice” AND “costs” or “benefits” or “effects” or “consequences” or “burden” or “impact” | **Patient population** #1  Mental disorders[MeSH Terms]  **Economic evaluation** #2  cost[Title/Abstract] OR costs[Title/Abstract] OR economic analysis[Title/Abstract] OR economic analyses[Title/Abstract] OR economic evaluation [Title/Abstract]OR costs and cost analysis[MeSH:noexp] OR cost-benefit analysis[MeSH Term] OR health care costs[MeSH] OR cost-of-illness[MeSH Term]  **Informal care*** #3  caregive* OR carer OR family carer OR home carer OR Informal care* OR Informal caregive* OR unpaid care* OR unprofessional care  **Patient, Family and societal perspective** #4  “patient perspective”[All Fields] OR “patient’s perspective”[All Fields] OR “societal”[All Fields] OR “social perspective”[All Fields] OR “family perspective”[All Fields] OR “patient’s cost”[All Fields] OR “patient’s costs”[All Fields] OR “service user’s cost” [All Fields] OR “service user’s costs” [All Fields] OR “family’s cost”[All Fields] OR “family’s costs”[All Fields] OR “out-of-pocket payment”[All Fields]  **All sector specific study** #5  #3 OR #4 |
| **Filters** |  | mental health” or “depressive disorder” or “conduct disorder” or “alcohol abuse” or “substance abuse” or “child abuse” |  |
| **Search limits** | Humans | Humans |  |
| **Language restrictions** | English, German, Dutch, Hungarian, Spanish | English | English, German, Dutch, Hungarian, Spanish |
| **Time restriction:** | PubMed: 09.05.2008 – 09.05.2018; PsycInfo: 07.05.2008 – 07.05.2018  NHS EED: 08.05.2008 – 08.05.2018 ; EconLit: 09.05.2008 – 09.05.2018  EMBASE: 18.05.2008 – 18.05.2018; DIRUM: 07.05.2008 – 07.05.2018 | 01.07.2012 | Past 10 years ("2008/06/18"[PDat] : "2018/06/15"[PDat]) |
| **Exclusion criteria:** | Editorials, letters, case reports and reviews, articles with other objects (i.e. not including cost calculations); studies based on results already published elsewhere and studies for which full texts were not available |  | Conference abstracts, reviews, editorials, letters, study protocols, full text not available |
| **Inclusion criteria:** |  |  | Full or partial economic evaluation, mental health, studies reporting original data, studies including services relevant for patient or family |

*Since criminal justice and education were covered by one work stream, one combined literature search was carried out.
