# Supplementary material for: Establishing a comprehensive list of mental health-related services and resource use items in Austria: A national-level, cross-sectoral country report for the PECUNIA project
Source: PLoS One. 2022 Jan 21;17(1):e0262091. doi: 10.1371/journal.pone.0262091 (PMC8782519; doi:10.1371/journal.pone.0262091)
Supplement: S2 Table — (DOCX) [file pone.0262091.s002.docx]

**Appendix 2**

**S2 Table:** Overview of the experts considered for recruitment for the expert survey within the specific sectors

| **Sectors** | **Considered national and regional experts** |
| --- | --- |
| **Health and social care** | The Austrian Federal Ministry for Labour, Social Affairs, Health and Consumer Protection (mainly in charge of the regulation, however, with cross-stakeholder structures at the federal state and local government levels) |
|  | National and regional stakeholders including experts from the health insurance funds, provincial governments and mental health care specific service providers |
|  | Mental health experts |
|  | Health services researchers |
|  | Public health experts |
| **Education** | The Austrian Federal Ministry of Education (in charge of schools and universities, its responsibility includes also school physicians and school social work) |
|  | Federal state education authorities (on the regional level, federal-state school physicians mainly coordinate health care provision in schools, school physicians provide health-related consultations to teachers and e.g. serial examinations of pupils) |
|  | School physicians with training in mental health care (to capture expertise at local level) |
| **Criminal justice** | Stakeholders working in forensic services and organizations providing specific services, such as treatment, counselling, assistance, housing, social rehabilitation and reintegration of mentally deranged offenders |
| **Patient, family and informal care** | Mental health disease-specific self-help groups, either specifically for the patient or for the patient and the family combined (available at the national, regional and local levels) |
|  | General self-help organization for informal caregivers (at national level) |
|  | Institutions in the social care sector that organize self-help groups for patients with mental diseases or provide relevant counselling via information centers |
