# Supplementary material for: Establishing a comprehensive list of mental health-related services and resource use items in Austria: A national-level, cross-sectoral country report for the PECUNIA project
Source: PLoS One. 2022 Jan 21;17(1):e0262091. doi: 10.1371/journal.pone.0262091 (PMC8782519; doi:10.1371/journal.pone.0262091)
Supplement: S3 Table — (DOCX) [file pone.0262091.s003.docx]

**Appendix 3**

**S3 Table: Design of the sector-specific expert surveys**

| **Health and social care sector** | | | | | |
| --- | --- | --- | --- | --- | --- |
| **Questions** | | | | | |
| 1. Is name of service and short definition clear to you? Please comment and/or make suggestions for improvement | 2. What percentage of persons diagnosed with a mental disorders use this service per year? | 3. What percentage of persons diagnosed with depression use this service per year? | 4.What percentage of persons diagnosed with schizophrenia use this service per year? | 5. What percentage of persons diagnosed with PTSD use this service per year? | 6. Please identify any missing services/resources and comment on why these are relevant |
| **Answer options** | | | | | |
| Free text | Very rare (<1%)  Occasionally (1-10%)  Frequent (>10%)  I don't know  Does not exist | Very rare (<1%)  Occasionally (1-10%)  Frequent (>10%)  I don't know  Does not exist | Very rare (<1%)  Occasionally (1-10%)  Frequent (>10%)  I don't know  Does not exist | Very rare (<1%)  Occasionally (1-10%)  Frequent (>10%)  I don't know  Does not exist | Free text |
| **Criminal justice sector** | | | | | |
| **Questions** | | | | | |
| 1. Please comment on the naming and the short definition of the item. Based on the name and short description, is it clear to you what the item entails? | 2. Do you think that this item is applicable to persons with mental disorders? | 3. Based on your insight, what percentage of costs incurred in (the national) criminal justice system in relation to persons diagnosed with mental disorders can be attributed to this item? | 4. From the economic perspective (based on frequency of occurrence and costliness),  which 8 items do you think are the most important? | 5. Do you think the list of items is complete? |  |
| **Answer options** | | | | | |
| Yes/No (if no, please suggest an alternative naming/definition that you believe is more clear) | Yes/No (if no, please explain) | A. <5%; B. 5-20%; C. >20%;  D. Do not know; E. Does not exist | Please rank only the 8 items in section 1 from 1 (most important) to 8 (less important). Do not mark anything for those items that you do not wish to select. | Yes/No (if no, please mention additional items) |  |
| **Education sector** | | | | | |
| **Questions** | | | | | |
| 1. Please comment on the naming and the short definition of the item. Based on the name and short description, is it clear to you what the item entails? | 2. Do you think that this item is applicable to students with mental disorders? | 3. Based on your insight, what percentage of costs incurred in the educational sector in relation to students diagnosed with mental disorders are attributed to this item? | 4. From the economic perspective (based on frequency of occurrence and costliness), which 5 items do you think are the most important? | 5. Do you think the list of items is complete? |  |
| **Answer options** | | | | | |
| Yes/no (if no, please suggest an alternative name/definition that you believe is more clear) | Yes/no (if no, please explain) | <5%  5-20%  >20%  Do not know  Does not exist | Please rank only the 5 most important items in section1 from 1 (most important) to 5 (less important). Do not mark anything for those items that you do not wish to select. | Yes/no (if no, please mention additional items that you think are/can be applicable) |  |
| **Patient, family and informal care sectors** | | | | | |
| **Questions** | | | | | |
| 1. Please comment on naming and short definition. Based on the name and short description, is it clear to you what the item entails? | 2.Based on your expertise, what percentage of persons diagnosed with mental disorders use the following service/goods per year? | 3.Based on your expertise, what percentage of persons diagnosed with depression use the following service/goods per year? | 4. Based on your expertise, what percentage of persons diagnosed with schizophrenia use the following service/goods per year? | 5.Based on your expertise, what percentage of persons diagnosed with PTSD use the following service/goods per year? | 6. Any other comments |
| **Answer options** | | | | | |
| Free text | <1%  1-10%  >10%  Does not exist | <1%  1-10%  >10%  Does not exist | <1%  1-10%  >10%  Does not exist | <1%  1-10%  >10%  Does not exist | Free text |
